# Re: Results of a randomized trial of treatment modalities in patients with low or early‑intermediate risk prostate cancer (PREFERE trial)

**DOI:** 10.1007/s00432-020-03499-x

**Published:** 2021-01-28

**Authors:** Andreas Boehle, Frank Kahmann, Thomas Oliver Henkel, Joerg Zimmermann, Stefan Machtens

**Affiliations:** 1 Department of Urology, University of Luebeck, Ratzeburger Allee 160, Luebeck, 23562 Germany; 2 Department of Urology, HELIOS Agnes-Karll Hospital, Am Hochkamp 21, Bad Schwartau, 23611 Germany; 3 MVZ Ihre Urologen GbR, Britzer Damm 63, 12347 Berlin, Germany; 4 Praxiszentrum Alstertal, Heegbarg 2, 22391 Hamburg, Germany; 5 Department of Urology, Marien-Krankenhaus, Dr.-Robert-Koch-Straße 18, 51465 Bergisch Gladbach, Germany

## Abstract

The authors of this “Letter to the Editors” express their major concern about selective and biased reporting in this paper.

Sir,

It is with concern that we read this publication.

In the abstract, the authors conclude on “an increased toxicity related to (PSI)”, neglecting the results as “treatment-related acute grade 3 toxicity was reported in 3 RP patients and 2 PSI patients”. Selectively emphasizing side effects of one treatment whilst neglecting even higher toxicity in another does not mirror scientific neutrality.

The results show that most patients accepted AS as a treatment strategy (84%). However, of all radical therapy options, PSI was the most accepted one (PSI 65%; RP 49%; EBRT 44%).

Furthermore, the lowest switch-over from one randomized radical therapy to another therapy was in PSI (change of randomized therapy from PSI 11%, EBRT 19%, and RP 19%). These data emphasize the relatively high acceptance of patients to PSI, but unfortunately remains uncommented by the authors.

CTCAE acute toxicity (< 12 months) grade 3 occurred in five patients, three in RP, and two in PSI, respectively. Figure 3a shows a higher percentage of overall toxicity (grades 1–3), a higher percentage of grade 3 toxicity, and a higher percentage of grade 2 toxicity in EBRT as compared to PSI.

CTCAE late toxicity (at 12 months) grade 3 occurred in 28 patients, 15 in RP, 9 in PSI, and 4 in EBRT, respectively. Figure 3b *of the original article* shows a higher percentage of overall toxicity (grades 1-3), a higher percentage of grade 3 toxicity, a higher percentage of grade 2 toxicity, and a higher percentage of grade 1 toxicity in EBRT as compared to PSI. Neglecting their own data, the authors state that “Interestingly, the preliminary data suggest an increased risk of toxicity after PSI compared with EBRT.” This statement is heavily biased toward EBRT and not supported by the facts.

The authors state that the study review revealed more quality-assurance issues in PSI than in RP and EBRT. This statement is not proven by any data given in the results. Furthermore, the authors neglect that in PSI, other than in EBRT and RP, a post-therapy quality control within 8 weeks is possible by so-called post-planning calculation. Notwithstanding the fact that post-planning documentation was missing/incomplete in 19%, an existing post-procedural quality-assurance in PSI cannot be compared to no post-procedural quality assurance at all, as in RP and EBRT.

Finally, with no data whatsoever reported on oncological outcomes, the authors state without proof that “oncological outcomes after PSI are unlikely to become superior to EBRT at the current state.” Again, we feel this statement is not supported by any facts and heavily biased toward EBRT.

Sincerely,

Andreas Boehle.

Frank Kahmann.

Thomas Oliver Henkel.

Joerg Zimmermann.

Stefan Machtens.

## Abbreviations

PSI: Permanent seed implantation
RP: Radical prostatectomy
EBRT: External beam radiation therapy
AS: Active surveillance
CTCAE: Common terminology criteria for adverse events

